# Liver fibrosis indices are related to diabetic peripheral neuropathy in individuals with type 2 diabetes

**DOI:** 10.1038/s41598-021-03870-z

**Published:** 2021-12-21

**Authors:** Kyuho Kim, Tae Jung Oh, Hyen Chung Cho, Yun Kyung Lee, Chang Ho Ahn, Bo Kyung Koo, Jae Hoon Moon, Sung Hee Choi, Hak Chul Jang

**Affiliations:** 1 Department of Internal Medicine, Seoul National University Bundang Hospital, 82 Gumi-ro, Bundang-gu, Seongnam-City, 13620 Republic of Korea; 2 Department of Internal Medicine, Seoul National University Boramae Medical Center, Seoul, Republic of Korea; 3 Department of Internal Medicine, Seoul National University College of Medicine, Seoul, Republic of Korea

**Keywords:** Diabetes complications, Liver fibrosis, Non-alcoholic fatty liver disease, Type 2 diabetes

## Abstract

The association between nonalcoholic fatty liver (NAFL) or liver fibrosis and diabetic peripheral neuropathy (DPN) has not been well studied. We aimed to investigate the association of NAFL or liver fibrosis indices and DPN in individuals with type 2 diabetes. In this observational study, we included 264 individuals with type 2 diabetes, and calculated non-alcoholic fatty liver disease (NAFLD) liver fat score, NAFLD fibrosis score, and Fibrosis-4 (FIB-4) index to evaluate the status of NAFLD or liver fibrosis. DPN was diagnosed when the Michigan Neuropathy Screening Instrument—Physical Examination score was ≥ 2.5. The NAFLD fibrosis score and FIB-4 index were significantly higher in individuals with DPN than in those without DPN. Logistic analyses showed that the NAFLD fibrosis score and FIB-4 index were associated with DPN after adjustment for covariates (adjusted odds ratio 1.474 and 1.961, respectively). In the subgroup analysis, this association was only significant in the group with a high NAFLD liver fat score (> − 0.640). Serum levels of fetuin-A, a hepatokine, were decreased in individuals with abnormal vibration perception or 10-g monofilament tests compared with their counterparts. The present study suggests that liver fibrosis might be associated with DPN in individuals with type 2 diabetes.

## Introduction

Nonalcoholic fatty liver disease (NAFLD) is the most prevalent chronic liver disease worldwide, affecting approximately 25% of the global population^1^. It encompasses a spectrum of diseases, extending from nonalcoholic fatty liver (NAFL) through nonalcoholic steatohepatitis (NASH), advanced fibrosis to cirrhosis, and end-stage liver disease. The natural history of NAFL is variable, with the majority of individuals having benign disease with steatosis without inflammation^2^. However, 40% of individuals with NAFL develop advanced fibrosis, which can result in cirrhosis^3^. The prevalence of NAFLD in individuals with type 2 diabetes is higher than that in the general population, ranging from 40 to 70%^2^. In addition, individuals with type 2 diabetes showed an increased risk of developing NASH, advanced fibrosis, and cirrhosis^4–7^. NAFLD in individuals with type 2 diabetes is associated with an increased risk of developing cardiovascular disease (CVD)^8,9^ and an increased risk of microvascular complications, such as nephropathy and retinopathy^10–12^.

Diabetic peripheral neuropathy (DPN) is the most common form of diabetic neuropathy and affects approximately 50% of individuals with diabetes^13^. Risk factors for DPN include hypertension, smoking, hyperglycaemia, diabetes duration, age, dyslipidaemia, and obesity, which are also known risk factors for CVD^14^. Considering that NAFLD, CVD, and DPN share risk factors and that NAFLD is associated with an increased risk of other microvascular complications, it is reasonable to hypothesize that individuals with diabetes and NAFL or liver fibrosis would also have a high risk for DPN.

In the present study, we examined the association of NAFL or liver fibrosis with DPN using noninvasive methods for evaluating NAFLD and liver fibrosis. In addition, we tested the association between DPN and fetuin-A, a hepatokine that is known to be elevated in individuals with NAFLD^15^.

## Results

### Clinical and biochemical characteristics of the study participants

The present study included the data of 264 individuals with type 2 diabetes but without chronic liver diseases; 38.2% had DPN. Body mass index (BMI), fasting plasma glucose (FPG), haemoglobin A_1c_ (HbA_1c_), and aspartate aminotransferase (AST) levels were significantly higher in individuals with DPN than in those without DPN (Table 1). The prevalence of suspected NAFLD based on NAFLD liver fat score > − 0.640 was compatible between individuals with and without DPN (73.3% vs. 69.3%, *p* = 0.493). However, both the NAFLD fibrosis score and Fibrosis-4 (FIB-4) index were significantly higher in individuals with DPN than in those without DPN.

**Table 1:** Demographics of study participants according to the presence of DPN.

Characteristics	Total (*n* = 264)	DPN (−) (*n* = 163)	DPN ( +) (*n* = 101)	*p* value
Male, *n* (%)	159 (60.2)	103 (63.2)	56 (55.4)	0.211
Age (years)	59.4 ± 9.2	58.8 ± 9.0	60.3 ± 9.4	0.212
Height (cm)	163.5 ± 8.9	163.9 ± 8.7	162.8 ± 9.1	0.312
Body weight (kg)	67.9 ± 11.7	67.3 ± 11.0	69.0 ± 12.6	0.243
BMI (kg/m^2^)	25.3 ± 3.4	24.9 ± 3.2	26.0 ± 3.8	0.016
Waist circumference (cm)	88.5 ± 8.8	87.9 ± 8.6	89.3 ± 9.0	0.208
Systolic BP (mmHg)	130.5 ± 14.0	130.4 ± 14.0	130.5 ± 14.0	0.932
Diastolic BP (mmHg)	74.9 ± 9.8	75.5 ± 10.2	74.0 ± 9.0	0.245
Diabetes duration (years)	11.3 ± 8.4	10.7 ± 8.4	12.2 ± 8.3	0.161
FPG (mmol/l)	7.6 ± 2.1	7.3 ± 1.5	8.0 ± 2.8	0.040
HbA_1c_ (mmol/mol)	55.5 ± 12.6	53.9 ± 11.1	58.0 ± 14.4	0.009
HbA_1c_ (%)	7.2 ± 1.2	7.1 ± 1.0	7.5 ± 1.3	0.009
Cholesterol (mmol/l)	4.0 ± 0.9	4.1 ± 0.9	3.9 ± 1.0	0.331
Triglyceride (mmol/l)^a^	1.3 ± 0.0	1.3 ± 0.0	1.4 ± 0.0	0.157
HDL cholesterol (mmol/l)^a^	1.2 ± 0.0	1.2 ± 0.0	1.2 ± 0.0	0.076
LDL cholesterol (mmol/l)	2.3 ± 0.7	2.4 ± 0.6	2.3 ± 0.7	0.212
Urea nitrogen (mmol/l)	5.9 ± 1.6	5.9 ± 1.5	5.9 ± 1.8	0.916
Creatinine (µmol/l)	70.7 ± 17.7	70.5 ± 17.6	70.7 ± 17.7	0.733
eGFR (mL min^−1^ [1.73 m]^−2^)	93.4 ± 21.9	94.8 ± 21.8	91.1 ± 21.9	0.177
AST (U/l)	28.5 ± 12.2	27.2 ± 10.3	30.5 ± 14.6	0.048
ALT (U/l)	29.3 ± 15.6	29.1 ± 15.3	29.6 ± 16.2	0.781
Insulin (pmol/l)	58.1 ± 30.8	56.7 ± 29.4	59.6 ± 33.0	0.456
HOMA-IR	2.8 ± 1.8	2.6 ± 1.5	3.0 ± 2.3	0.097
Abnormal 10-g monofilament test^b^, *n* (%)	18 (6.8)	4 (2.5)	14 (13.9)	< 0.001
MNSI-Q	2.1 ± 2.0	1.7 ± 1.9	2.8 ± 2.1	< 0.001
MNSI-PE	2.1 ± 1.2	1.3 ± 0.6	3.5 ± 0.7	< 0.001
Abnormal appearance, *n* (%)	93 (35.2)	10 (6.1)	83 (82.2)	< 0.001
Ulceration, *n* (%)	1 (0.4)	0 (0.0)	1 (1.0)	0.203
Absent ankle reflexes, *n* (%)	18 (6.8)	4 (2.5)	14 (13.9)	< 0.001
Absent vibration perception, *n* (%)	52 (19.7)	19 (11.7)	33 (32.7)	< 0.001
**Smoking status, *n*** **(%)**				0.131
Never smoker	124 (47.0)	69 (42.3)	55 (54.5)	
Ex-smoker	86 (32.6)	56 (34.4)	30 (29.7)	
Current smoker	54 (20.5)	38 (23.3)	16 (15.8)	
Alcohol, *n* (%)	102 (38.6)	62 (38.0)	40 (39.6)	0.799
Exercise, *n* (%)	180 (68.2)	110 (67.5)	70 (69.3)	0.757
Hypertension, *n* (%)	145 (54.9)	86 (52.8)	59 (58.4)	0.369
Dyslipidaemia, *n* (%)	174 (65.9)	104 (63.8)	70 (69.3)	0.359
Insulin therapy, *n* (%)	50 (18.9)	27 (16.6)	23 (22.8)	0.211
Lipid-lowering agent, *n* (%)	203 (76.9)	124 (76.1)	79 (78.2)	0.688
NAFLD liver fat score	0.04 ± 1.24	− 0.04 ± 1.20	0.15 ± 1.29	0.224
NAFLD liver fat score > − 0.640, *n* (%)	187 (70.8)	113 (69.3)	74 (73.3)	0.493
NAFLD fibrosis score	− 0.97 ± 1.11	− 1.11 ± 1.08	− 0.75 ± 1.14	0.010
NAFLD fibrosis score > 0.676, *n* (%)	16 (6.1)	5 (3.1)	11 (10.9)	0.010
FIB-4 index	1.43 ± 0.68	1.34 ± 0.59	1.58 ± 0.79	0.009
FIB-4 index ≥ 1.3^c^, *n* (%)	106 (40.2)	57 (35.0)	49 (48.5)	0.029

Data are expressed as the mean ± standard deviation (SD) or geometric mean ± geometric SD or number (%).

*DPN* diabetic peripheral neuropathy, *BMI* body mass index, *BP* blood pressure, *FPG* fasting plasma glucose, *HbA*_*1c*_ haemoglobin A_1c_, *HDL* high-density lipoprotein, *LDL* low-density lipoprotein, *eGFR* estimated glomerular filtration rate, *AST* aspartate aminotransferase, *ALT* alanine aminotransferase, *HOMA-IR* homeostatic model assessment-insulin resistance, *MNSI-Q* michigan neuropathy screening instrument-questionnaire, *MNSI-PE* michigan neuropathy screening instrument-physical examination, *NAFLD* nonalcoholic fatty liver disease, *FIB-4* fibrosis-4.

^a^Variable was natural log-transformed before statistical analysis and expressed as geometric mean ± geometric SD.

^b^Abnormal 10-g monofilament test was defined as a 10-g monofilament score ˂ 7 on either side.

^c^For individuals aged ≥ 65 years, a cut-off of 2.0 was used. *p* value for χ^2^ test or *t* test.

We stratified individuals by NAFLD liver fat score (≤ − 0.640 or > − 0.640) and the presence of DPN. Compared with individuals with a low NAFLD liver fat score (≤ − 0.640), individuals with a high NAFLD liver fat score (> − 0.640) were more obese and had higher blood pressure (BP), triglyceride levels, AST, alanine aminotransferase (ALT), and homeostatic model assessment-insulin resistance (HOMA-IR) (Table 2). However, there were no significant differences in neuropathy examination results between individuals with a low NAFLD liver fat score (≤ − 0.640) and those with a high NAFLD liver fat score (> − 0.640). In contrast to the subgroup with a low NAFLD liver fat score (≤ − 0.640), the NAFLD fibrosis score and FIB-4 index were significantly higher in individuals with DPN than in those without DPN among individuals with a high NAFLD liver fat score (> − 0.640).

**Table 2:** Clinical and biochemical characteristics of individuals with type 2 diabetes according to the NAFLD liver fat score and the presence of DPN.

Characteristic	NAFLD liver fat score ≤ -0.640				NAFLD liver fat score > − 0.640				*p* value^d^
	Total (*n* = 77)	DPN (−) (*n* = 50)	DPN ( +) (*n* = 27)	*p* value	Total (*n* = 187)	DPN (−) (*n* = 113)	DPN ( +) (*n* = 74)	*p* value	
Male, *n* (%)	47 (61.0)	30 (60.0)	17 (63.0)	0.799	112 (59.9)	73 (64.6)	39 (52.7)	0.104	0.863
Age (years)	58.2 ± 10.7	57.4 ± 10.2	59.7 ± 11.5	0.367	59.9 ± 8.4	59.5 ± 8.3	60.5 ± 8.5	0.420	0.222
Height (cm)	164 ± 8.5	164.2 ± 8.1	163.7 ± 9.4	0.791	163.2 ± 9.0	163.8 ± 9.0	162.4 ± 9.0	0.326	0.506
Body weight (kg)	62.3 ± 9.1	61.8 ± 9.1	63.2 ± 9.1	0.516	70.2 ± 11.8	69.7 ± 11.0	71.1 ± 13.0	0.427	< 0.001
BMI (kg/m^2^)	23.0 ± 2.6	22.8 ± 2.6	23.5 ± 2.4	0.218	26.3 ± 3.3	25.9 ± 2.9	26.9 ± 3.8	0.060	< 0.001
Waist circumference (cm)	83.4 ± 7.3	82.3 ± 7.5	85.6 ± 6.4	0.058	90.5 ± 8.5	90.4 ± 7.9	90.7 ± 9.4	0.817	< 0.001
SBP (mmHg)	126.4 ± 12.5	127.0 ± 12.8	125.1 ± 12.0	0.537	132.1 ± 14.2	131.9 ± 14.3	132.5 ± 14.2	0.772	0.002
DBP (mmHg)	72.4 ± 8.3	73.3 ± 8.4	70.7 ± 8.0	0.191	76.0 ± 10.2	76.5 ± 10.8	75.3 ± 9.1	0.434	0.006
Diabetes duration (years)	11.4 ± 9.5	10.2 ± 9.4	13.5 ± 9.4	0.140	11.3 ± 7.9	11.0 ± 8.0	11.8 ± 7.8	0.518	0.968
FPG (mmol/l)	7.6 ± 1.9	7.3 ± 1.6	8.2 ± 2.2	0.042	7.6 ± 2.2	7.4 ± 1.5	7.9 ± 3.0	0.167	0.963
HbA_1c_ (mmol/mol)	54.3 ± 12.3	53.2 ± 12.1	56.4 ± 12.5	0.273	55.9 ± 12.8	54.2 ± 10.7	58.6 ± 15.0	0.019	0.346
HbA_1c_ (%)	7.1 ± 1.1	7.0 ± 1.1	7.3 ± 1.1	0.279	7.3 ± 1.2	7.1 ± 1.0	7.5 ± 1.4	0.020	0.349
Cholesterol (mmol/l)	4.1 ± 0.9	4.3 ± 1.0	3.9 ± 0.7	0.075	4.0 ± 0.9	4.0 ± 0.8	4.0 ± 1.1	0.981	0.165
Triglyceride (mmol/l)^a^	1.1 ± 0.0	1.1 ± 0.0	1.2 ± 0.0	0.632	1.4 ± 0.0	1.3 ± 0.0	1.5 ± 0.0	0.252	< 0.001
HDL cholesterol (mmol/l)^a^	1.2 ± 0.0	1.2 ± 0.0	1.3 ± 0.0	0.979	1.2 ± 0.0	1.2 ± 0.0	1.1 ± 0.0	0.045	0.198
LDL cholesterol (mmol/l)	2.4 ± 0.6	2.5 ± 0.7	2.2 ± 0.6	0.112	2.3 ± 0.7	2.3 ± 0.6	2.3 ± 0.7	0.655	0.394
Urea nitrogen (mmol/l)	5.8 ± 1.3	5.8 ± 1.1	5.9 ± 1.4	0.591	5.9 ± 1.8	5.9 ± 1.7	5.9 ± 2.0	0.897	0.701
Creatinine (µmol/l)	70.7 ± 17.7	70.7 ± 17.6	70.7 ± 17.7	0.916	70.7 ± 17.7	70.7 ± 17.6	70.7 ± 17.7	0.799	0.122
eGFR (mL min^-1^ [1.73 m]^-2^)	97.2 ± 21.0	98.2 ± 21.9	95.3 ± 19.3	0.559	91.8 ± 22.1	93.3 ± 21.7	89.5 ± 22.7	0.256	0.070
AST (U/l)	23.2 ± 5.6	23.4 ± 6.3	22.8 ± 4.1	0.666	30.7 ± 13.4	28.9 ± 11.2	33.3 ± 16.0	0.041	< 0.001
ALT (U/l)	20.6 ± 7.7	20.7 ± 8.5	20.4 ± 6.1	0.875	32.9 ± 16.6	32.8 ± 16.2	33.0 ± 17.4	0.933	< 0.001
Insulin (pmol/l)	39.5 ± 16.5	39.5 ± 17.9	39.5 ± 12.9	0.950	65.3 ± 32.3	64.6 ± 30.1	67.5 ± 34.4	0.572	< 0.001
HOMA-IR	1.9 ± 0.9	1.8 ± 0.9	2.0 ± 1.0	0.273	3.1 ± 2.0	3.0 ± 1.5	3.4 ± 2.5	0.194	< 0.001
Abnormal 10-g monofilament test^b^, *n* (%)	2 (2.6)	0 (0.0)	2 (7.4)	0.051	16 (8.6)	4 (3.5)	12 (16.2)	0.002	0.081
MNSI-Q	1.7 ± 1.8	1.4 ± 1.7	2.3 ± 1.8	0.043	2.3 ± 2.1	1.8 ± 1.9	3.0 ± 2.2	< 0.001	0.054
MNSI-PE	2.1 ± 1.2	1.3 ± 0.5	3.4 ± 0.8	< 0.001	2.2 ± 1.3	1.3 ± 0.6	3.5 ± 0.7	< 0.001	0.428
Abnormal appearance, *n* (%)	23 (29.9)	3 (6.0)	20 (74.1)	< 0.001	70 (37.4)	7 (6.2)	63 (85.1)	< 0.001	0.242
Ulceration, *n* (%)	0 (0.0)	0 (0.0)	0 (0.0)	NA	1 (0.5)	0 (0.0)	1 (1.4)	0.215	0.520
Absent ankle reflexes, *n* (%)	4 (5.2)	1 (2.0)	3 (11.1)	0.086	14 (7.5)	3 (2.7)	11 (14.9)	0.002	0.502
Absent vibration perception, *n* (%)	13 (16.9)	4 (8.0)	9 (33.3)	0.005	39 (20.9)	15 (13.3)	24 (32.4)	0.002	0.461
**Smoking status, *n*** **(%)**				0.763				0.133	0.101
Never smoker	37 (48.1)	23 (46.0)	14 (51.9)		87 (46.5)	46 (40.7)	41 (55.4)		
Ex-smoker	19 (24.7)	12 (24.0)	7 (25.9)		67 (35.8)	44 (38.9)	23 (31.1)		
Current smoker	21 (27.3)	15 (30.0)	6 (22.2)		33 (17.6)	23 (20.4)	10 (13.5)		
Alcohol, *n* (%)	25 (32.5)	15 (30.0)	10 (37.0)	0.529	77 (41.2)	47 (41.6)	30 (40.5)	0.886	0.187
Exercise, *n* (%)	54 (70.1)	32 (64.0)	22 (81.5)	0.110	126 (67.4)	78 (69.0)	48 (64.9)	0.553	0.663
Hypertension, *n* (%)	15 (19.5)	10 (20.0)	5 (18.5)	0.876	130 (69.5)	76 (67.3)	54 (73.0)	0.406	< 0.001
Dyslipidaemia, *n* (%)	35 (45.5)	21 (42.0)	14 (51.9)	0.407	139 (74.3)	83 (73.5)	56 (75.7)	0.733	< 0.001
Insulin therapy, *n* (%)	14 (18.2)	8 (16.0)	6 (22.2)	0.499	36 (19.3)	19 (16.8)	17 (23.0)	0.296	0.840
Lipid-lowering agent, *n* (%)	49 (63.6)	29 (58.0)	20 (74.1)	0.162	154 (82.4)	95 (61.7)	59 (38.3)	0.446	0.001
NAFLD fibrosis score	− 1.00 ± 1.13	− 1.15 ± 1.20	− 0.73 ± 0.93	0.120	− 0.96 ± 1.11	− 1.10 ± 1.02	− 0.76 ± 1.22	0.041	0.806
NAFLD fibrosis score > − 0.676, *n* (%)	4 (5.2)	3 (6.0)	1 (3.7)	0.665	12 (6.4)	2 (1.8)	10 (13.5)	0.001	0.705
FIB-4 index	1.41 ± 0.65	1.37 ± 0.69	1.48 ± 0.59	0.487	1.44 ± 0.69	1.33 ± 0.54	1.62 ± 0.85	0.010	0.702
FIB-4 index ≥ 1.3, *n* (%)^c^	29 (37.7)	17 (34.0)	12 (44.4)	0.367	77 (41.2)	40 (35.4)	37 (50.0)	0.047	0.597

Data are expressed as the mean ± standard deviation (SD) or geometric mean ± geometric SD or number (%).

*DPN* diabetic peripheral neuropathy, *BMI* body mass index, *BP* blood pressure, *FPG* fasting plasma glucose, *HbA*_*1c*_ haemoglobin A_1c_, *HDL* high-density lipoprotein, *LDL* low-density lipoprotein, *eGFR* estimated glomerular filtration rate, *AST* aspartate aminotransferase, *ALT* alanine aminotransferase, *HOMA-IR* homeostatic model assessment-insulin resistance, *MNSI-Q* michigan neuropathy screening instrument-questionnaire, *MNSI-PE* michigan neuropathy screening instrument-physical examination, *NAFLD* nonalcoholic fatty liver disease, *FIB-4* fibrosis-4.

^a^Variable was natural log-transformed before statistical analysis and expressed as geometric mean ± geometric SD.

^b^Abnormal 10-g monofilament test was defined as a 10-g monofilament score ˂ 7 on either side.

^c^For individuals aged ≥ 65 years, a cut-off of 2.0 was used.

^d^Comparison between NAFLD liver fat score ≤ − 0.640 group and NAFLD liver fat score > − 0.640 group. *p* value for χ^2^ test or *t* test.

### Association of NAFLD fibrosis score and FIB-4 index with DPN

Logistic regression analyses showed that the NAFLD liver fat score was not associated with DPN. However, the NAFLD fibrosis score and FIB-4 index were significantly associated with DPN: adjusted odds ratio (aOR) 1.474 (95% confidence interval [CI] 1.055, 2.058), and aOR 1.961 (95% CI 1.209, 3.183), respectively (Table 3). In the subgroup analysis, this association was only observed in individuals with a high NAFLD liver fat score (> − 0.640). The aORs for the NAFLD fibrosis score and FIB-4 index were 1.501 (95% CI 1.006, 2.239) and 2.272 (95% CI 1.271, 4.059), respectively.

**Table 3:** ORs between NAFLD liver fat score, NAFLD fibrosis score, FIB-4 index, and DPN.

	Total population			NAFLD liver fat score ≤ − 0.640			NAFLD liver fat score > − 0.640		
	OR	95% CI	*p* value	OR	95% CI	*p* value	OR	95% CI	*p* value
**NAFLD liver fat score**									
Model 1	1.132	0.926, 1.384	0.225	0.964	0.328, 2.838	0.947	1.170	0.879, 1.558	0.282
Model 2	1.028	0.814, 1.300	0.815	0.856	0.275, 2.666	0.788	1.085	0.793, 1.483	0.611
Model 3	0.910	0.677, 1.224	0.533	0.485	0.132, 1.775	0.274	0.996	0.677, 1.465	0.983
**NAFLD fibrosis score**									
Model 1	1.445	1.116, 1.872	0.005	1.413	0.911, 2.191	0.122	1.354	1.004, 1.827	0.047
Model 2	1.389	1.003, 1.923	0.048	1.451	0.783, 2.689	0.237	1.386	0.944, 2.035	0.096
Model 3	1.474	1.055, 2.058	0.023	1.681	0.824, 3.431	0.153	1.501	1.006, 2.239	0.047
**FIB-4 index**									
Model 1	1.689	1.156, 2.467	0.007	1.288	0.634, 2.615	0.484	1.879	1.185, 2.978	0.007
Model 2	1.753	1.106, 2.781	0.017	1.351	0.508, 3.596	0.546	2.028	1.166, 3.527	0.012
Model 3	1.961	1.209, 3.183	0.006	1.840	0.617, 5.491	0.274	2.272	1.271, 4.059	0.006

Data are presented as odds ratio (OR) and 95% confidence interval (CI). Model 1 is unadjusted. Model 2 is adjusted for sex, age, body mass index (BMI), systolic blood pressure (BP), and diabetes duration. Model 3 is additionally adjusted for haemoglobin A_1c_ (HbA_1c_), low-density lipoprotein (LDL) cholesterol, and homeostatic model assessment-insulin resistance (HOMA-IR). NAFLD, nonalcoholic fatty liver disease; FIB-4, fibrosis-4; DPN, diabetic peripheral neuropathy.

### Association between fetuin-A and DPN features

**Figure 1:**
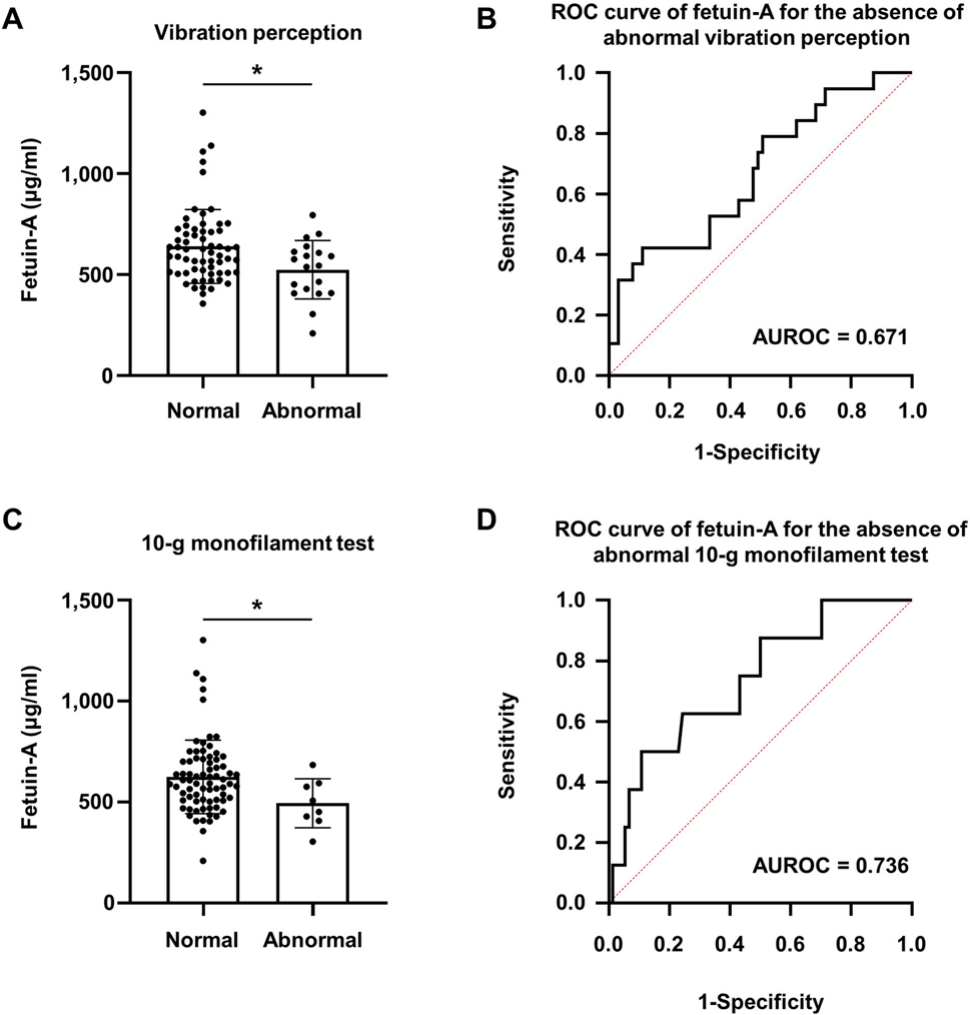
Serum fetuin-A levels and ROC curves for the detection of abnormalities in DPN examination in individuals with a high NAFLD liver fat score (≥ − 0.640). (**A**) Normal (*n* = 63) and abnormal (*n* = 19) vibration perception. (**B**) ROC curve of fetuin-A for the absence of abnormal vibration perception. (**C**) Normal (*n* = 74) and abnormal (*n* = 8) 10-g monofilament tests. (**D**) ROC curve of fetuin-A for the absence of an abnormal 10-g monofilament test. *p < 0.05. Data are the mean ± standard deviation. AUROC, area under the ROC curve; DPN, diabetic peripheral neuropathy; NAFLD, nonalcoholic fatty liver disease; ROC, receiver operating characteristic.

Among individuals with a high NAFLD liver fat score (> − 0.640), serum fetuin-A levels were 613.5 ± 181.0 µg/ml in individuals without DPN and 611.3 ± 182.7 µg/ml in those with DPN (*p* = 0.956). Serum fetuin-A levels were significantly lower in individuals with abnormal vibration perception (542.2 ± 144.9 µg/ml vs. 639.0 ± 183.0 µg/ml, *p* = 0.014) and in those with an abnormal 10-g monofilament test (494.2 ± 121.0 µg/ml vs. 625.2 ± 182.1 µg/ml, *p* = 0.029) compared with their counterparts. The area under the receiver operating characteristic curve (AUROC) of the fetuin-A levels for the absence of abnormal vibration perception was 0.671 (95% CI 0.531, 0.811) and for the absence of abnormal 10-g monofilament test was 0.736 (95% CI 0.561, 0.911) (Fig. 1).

### Discrimination power of liver fibrosis indices and fetuin-A for DPN

Supplementary Table S1 shows the discrimination power and various cut-off values of the NAFLD fibrosis score, FIB-4 index, and fetuin-A for DPN. Overall, the performance was not sufficient for use as a diagnostic tool for DPN.

## Discussion

In this cross-sectional study, there was a lack of significant association between NAFLD liver fat score and DPN, but liver fibrosis indices such as NAFLD fibrosis index and FIB-4 index were higher in individuals with DPN than in individuals without DPN. In addition, even after adjustment for known DPN risk factors, the NAFLD fibrosis score and FIB-4 index were independently associated with DPN.

Previous studies have shown conflicting results regarding the association between NAFLD and DPN. Mantovani et al.^16^ used ultrasonography for the diagnosis of NAFLD, and they used the Michigan Neuropathy Screening Instrument (MNSI) method and a vibration perception threshold (VPT) assessment for the diagnosis of DPN. They showed a positive association between NAFLD and DPN in Italian individuals with type 1 diabetes (mean age 43.4 years, mean HbA_1c_ 8.0%, and median diabetes duration 17 years). Lv et al.^17^ used ultrasonography for the diagnosis of NAFLD, and diagnosed DPN based on physical examination. They showed a negative association between NAFLD and DPN in hospitalised Chinese individuals with type 2 diabetes (mean age 63.4 years, mean HbA_1c_ 8.7%, and mean diabetes duration 9.6 years). Kim et al.^18^ used ultrasonography for the diagnosis of NAFLD, and used a nerve conduction study, a current perception threshold test, and physical examination for the diagnosis of DPN. They showed no association between NAFLD and DPN in Korean individuals with type 2 diabetes (mean age 57.7 years, mean HbA_1c_ 8.4%, and mean diabetes duration 6.2 years), which was consistent with our results. Potential explanations for these differences are the different characteristics of participants in each study and different diagnostic criteria for DPN. Otherwise, considering that the majority of individuals with NAFLD in previous studies were estimated to have NAFL, which is an early stage of NAFLD, other medical conditions or more severe stages of NAFLD might be more important contributors to DPN than NAFL per se.

A previous cohort study reported that an elevated lower-limb vibration perception threshold was associated with markers of liver fibrosis, such as the NAFLD fibrosis score, and with liver stiffness measurement in individuals with type 2 diabetes^19^. Our study is consistent with previous observations, and we further found that the association between DPN and liver fibrosis indices was only significant in individuals with a high NAFLD liver fat score (> − 0.640). This can be explained by increased vulnerability of the liver to injuries such as oxidative stress or cytokines, as reflected by higher BMI, AST, ALT, and HOMA-IR levels in individuals with a high NAFLD liver fat score (> − 0.640) compared with those with low NAFLD liver fat score (≤ − 0.640).

The ‘multiple hit hypothesis’ suggests that multiple insults might be generated in individuals with type 2 diabetes due to altered inter-organ crosstalk between the intestine, adipose tissue, skeletal muscle, liver, and pancreas, and that these insults would synergistically result in the development and progression of NAFLD^20^. During the development of NAFLD, hypercaloric diets can induce intestinal dysbiosis and excess fat storage in adipose tissue, skeletal muscle, and liver, which result in inflammation and insulin resistance. Insulin resistance results in hyperglycaemia and hyperinsulinaemia. During the progression of NAFLD, glucolipotoxicity increases reactive oxygen species (ROS) generation and endoplasmic reticulum stress, resulting in cell death. Together, these dead cells combined with infiltrated inflammatory cells in the liver, free fatty acids, intestine-derived lipopolysaccharides, and transforming growth factor (TGF)-β from Kupffer cells activate hepatic stellate cells (HSCs). Activated HSCs increase the extracellular matrix, leading to liver fibrosis. Among these insults related to the progression of NAFLD, hyperglycaemia, insulin resistance, oxidative stress, and inflammation are also involved in the pathogenesis of DPN^21^.

Advanced glycation end products (AGEs) are implicated in the pathogenesis of DPN. The formation of AGEs increases under chronic hyperglycaemia in diabetes. Interaction of AGEs with their receptors (RAGEs) activates intracellular signalling pathways and increases oxidative stress and inflammation, ultimately resulting in neuronal injuries^22,23^. Interestingly, patients with NASH exhibited higher hepatic and serum glyceraldehyde-derived AGEs levels than those with simple steatosis or healthy controls^24^. In addition, glyceraldehyde-derived AGEs increase ROS generation and upregulate fibrogenic genes such as *α-smooth muscle actin*, *TGF-β1*, and *collagen type Iα2* in human hepatic stellate cell line in vitro^25^. These results suggest that glyceraldehyde-derived AGEs may contribute to the pathogenesis of NASH. Considering the potential role of AGEs and RAGEs in the pathogenesis of both DPN and NAFLD, our finding that the NAFLD fibrosis score and FIB-4 index were associated with DPN appears reasonable.

The progression of NAFLD alters the secretion of hepatokines such as fetuin-A, fetuin-B, and dipeptidyl peptidase-4^26,27^, and we evaluated an association between fetuin-A and DPN. Serum fetuin-A levels were negatively associated with abnormal vibration perception and abnormal 10-g monofilament tests. Considering a previous study that showed TGF-β1 signalling suppression by fetuin-A^28^, and a previous study that showed high TGF-β1 levels in individuals with DPN^29^, our results seem to suggest a possibility of link between fetuin-A and DPN. Although, fetuin-A cannot be used as a diagnostic tool for DPN, this link suggests the possibility of loss of protection sensation.

This study has several limitations. First, it cannot establish a causal relationship because of its cross-sectional nature. Second, liver biopsy, the gold standard method for the diagnosis of NAFLD and liver fibrosis, was not performed. Third, neurophysiological studies were not used for the diagnosis of DPN. Despite these limitations, this study provides valuable insight implying that the progression of NAFL to liver fibrosis might affect the development of DPN and suggests the possible role of fetuin-A in specific feature of DPN, a loss of protection sensation.

In conclusion, liver fibrosis might be associated with DPN in individuals with type 2 diabetes and suspected NAFLD. Notably, this association was independent of previously known risk factors. The present study suggests the need for special attention to DPN in individuals with type 2 diabetes and NAFLD, especially those with a high NAFLD fibrosis score or FIB-4 index. Future studies to investigate the molecular mechanism of the association between liver fibrosis and DPN are necessary.

## Methods

### Study population

**Figure 2:**
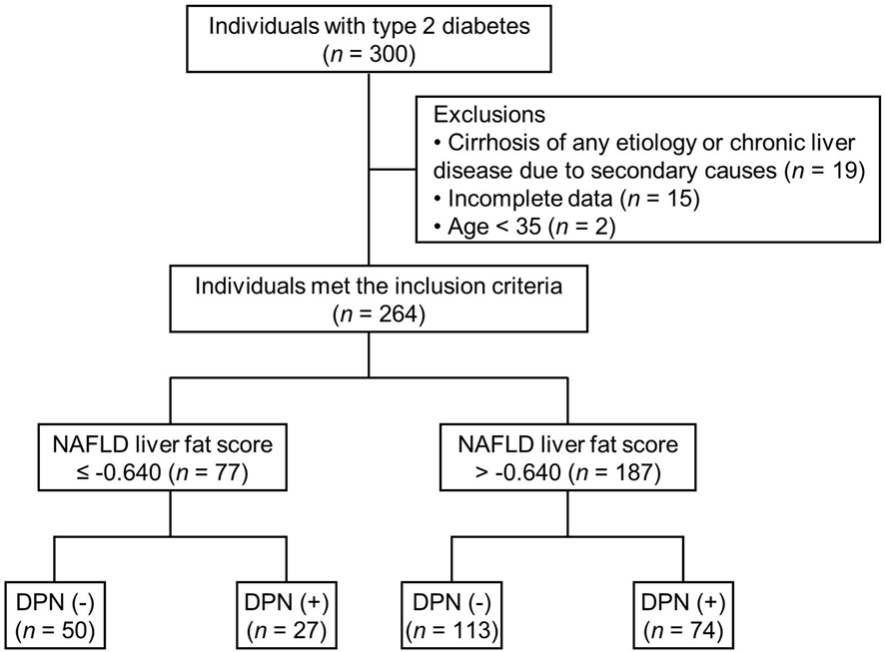
Flow chart of the selection of individuals for the analysis. Among 264 individuals with type 2 diabetes, individuals with suspected NAFLD (*n* = 187) were selected based on the NAFLD liver fat score. They were divided into 2 groups according to the presence of DPN. DPN, diabetic peripheral neuropathy; NAFLD, nonalcoholic fatty liver disease.

A prospective observational study is ongoing to discover reliable screening tools and biomarkers for DPN. The inclusion criteria were age ≥ 19 years, diagnosis of type 2 diabetes, and no change in glucose-lowering drugs in the last 3 months. The exclusion criteria were stage 4 or 5 chronic kidney disease (estimated glomerular filtration rate [eGFR] < 30 mL min^−1^ [1.73 m]^−2^), pregnancy, and severe diabetic foot ulcers or previous amputation. This is a subset study analysing data from individuals who were enrolled during the initial 3-year period (January 2017 to January 2020). We recruited 300 individuals with type 2 diabetes from Seoul National University Bundang Hospital (SNUBH), a tertiary academic hospital. In the present study, the following individuals were excluded: (1) individuals (*n* = 19) with cirrhosis of any etiology or chronic liver disease due to excessive alcohol consumption (alcohol consumption > 30 g/day for men and > 20 g/day for women) or viral hepatitis based on a medical history and medications; (2) individuals (*n* = 15) with incomplete data needed to calculate the NAFLD liver fat score, NAFLD fibrosis score, or FIB-4 index; and (3) individuals (*n* = 2) aged under 35 years due to poor performance of NAFLD fibrosis score and FIB-4 index for a diagnosis of liver fibrosis in those aged ≤ 35 years^30^. The remaining 264 individuals with type 2 diabetes were included in the final analysis (Fig. 2). The study was approved by the Institutional Review Board of SNUBH (no. B-2012-657-106), and was performed in accordance with relevant guidelines and regulations. All participants provided written informed consent.

### Anthropometric and biochemical analyses

Anthropometric indices and neurologic tests were measured by a well-trained research nurse. BMI was calculated as weight (kg) divided by the square of the height (m). Waist circumference was measured at the midpoint between the margin of the lowest rib and the iliac crest. Systolic BP and diastolic BP were measured by an electronic blood pressure metre after 10 min of rest in a sitting position. Alcohol consumption was assessed by two questions from the Alcohol Use Disorders Identification Test-Consumption: (1) the usual frequency of drinking, (2) the typical quantity of drinking^31^. We defined drinkers as those who drink any alcoholic beverage more than once a month. Smoking status was classified as never smoker (< 100 cigarettes in lifetime and currently a nonsmoker), ex-smoker (≥ 100 cigarettes in lifetime and currently a nonsmoker), and current smoker (≥ 100 cigarettes in lifetime and currently a smoker). Positive exercise was defined as exercising for > 150 min/week. Blood samples were collected after an overnight fast. FPG levels were measured by the hexokinase method, and HbA_1c_ levels were measured by high-performance liquid chromatography (Bio-Rad, Hercules, CA, USA). Serum insulin levels were measured by immunoradiometric assay (DIAsource, Nivelles, Belgium). Total cholesterol, triglyceride, high-density lipoprotein (HDL) cholesterol, and LDL cholesterol were measured by enzymatic colorimetric assay. Liver function tests, including AST and ALT, and renal function tests were measured by the protocol of the central laboratory of SNUBH. HOMA-IR was calculated using the following formula^32^: HOMA-IR = (fasting insulin [μIU/ml] × FPG [mg/dl]/405). Among individuals with a high NAFLD liver fat score (> -0.640), serum fetuin-A levels of 41 individuals with DPN and 41 individuals without DPN were measured using commercial enzyme-linked immunosorbent assay (ELISA) kits (R&D Systems, no. DFTA00, Minneapolis, MN, USA).

### Assessment of microvascular complications of diabetes

DPN was assessed using the MNSI, which includes two separate assessments: a 15-item self-administered questionnaire (MNSI-Q) and a lower extremity physical examination (MNSI-PE)^33^. The MNSI-PE is scored for abnormalities of foot appearance such as deformities, dry skin, calluses, infections and fissures (normal = 0, abnormal = 1), ulceration (absent = 0, present = 1), vibration perception at great toe (absent = 1, reduced = 0.5, present = 0), and ankle reflexes (absent = 1, present with reinforcement = 0.5, present = 0). The total possible score is 8 points for both feet. DPN was diagnosed when the MNSI-PE score was ≥ 2.5, based on prior studies^34,35^. A 10-g monofilament test was considered abnormal when an individual had a sensation of fewer than seven points on one of the two feet^36^. Abnormal appearance was defined as the presence of any abnormality except ulceration, as ulceration was defined separately. Ankle reflexes were tested using a tendon hammer at the Achilles tendon. Abnormal vibration perception was defined as the absence of vibration perception on either side of the great toe using a 128-Hz tuning fork. A trained nurse performed all neurologic examinations.

### Noninvasive methods for evaluating NAFLD and liver fibrosis

The NAFLD liver fat score was calculated according to the following formula: − 2.89 + 1.18 × metabolic syndrome (yes = 1, no = 0) + 0.45 × type 2 diabetes (yes = 2, no = 0) + 0.15 × fasting serum insulin (IU/l) + 0.04 × AST (U/l) − 0.94 × AST/ALT. A NAFLD liver fat score > − 0.640 was used to identify suspected NAFLD according to a previous report that a score > − 0.640 detected NAFLD with a sensitivity of 86% and specificity of 71%^37^. The NAFLD fibrosis score was calculated according to the following formula: − 1.675 + 0.037 × age (years) + 0.094 × BMI (kg/m^2^) + 1.13 × impaired fasting glucose or diabetes (yes = 1, no = 0) + 0.99 × AST/ALT − 0.013 × platelets (10^9^/l) − 0.66 × albumin (g/dl). A NAFLD fibrosis score > 0.676 was used to identify liver fibrosis^38^. The FIB-4 index was calculated according to the following formula: (age [years] × AST [U/l])/(platelets [10^9^/l] × ALT^1/2^ [U/l]). A FIB-4 index ≥ 1.3 was used to identify liver fibrosis^39^. However, for individuals aged ≥ 65 years, a FIB-4 index ≥ 2.0 was used to identify liver fibrosis as previously reported^30^.

### Statistical analysis

Data were expressed as the mean ± standard deviation (SD) or number (%). Variables with a nonnormal distribution were log-transformed prior to analysis. Comparisons of continuous variables between individuals with and without DPN were performed using Student’s unpaired *t* tests. Categorical variables were compared using χ^2^ tests. The associations between the presence of DPN and NAFLD liver fat score, NAFLD fibrosis score, and FIB-4 index were analysed using logistic regression models. Multivariable logistic regression analysis was performed including known risk factors for DPN. The prediction performance of liver fibrosis indices and serum fetuin-A levels for DPN and for the absence of abnormal vibration perception or absence of abnormal 10-g monofilament test was assessed by analysing receiver operating characteristic (ROC) curves, and the AUROC was calculated. Based on various cut-off values, we calculated the sensitivity, specificity, positive predictive value, and negative predictive value. In all cases, *p* < 0.05 was considered statistically significant. Statistical analyses were performed using IBM SPSS version 25.0 (IBM, Armonk, NY, USA). Figures were drawn using GraphPad Prism software (version 9.1.2; GraphPad Software Inc., San Diego, CA, USA).

## Supplementary Information

Supplementary Table S1.
