# Supplementary material for: Liver fibrosis indices are related to diabetic peripheral neuropathy in individuals with type 2 diabetes
Source: Sci Rep. 2021 Dec 21;11:24372. doi: 10.1038/s41598-021-03870-z (PMC8692472; doi:10.1038/s41598-021-03870-z)
Supplement: Supplementary file 1 — Supplementary Table S1. [file 41598_2021_3870_MOESM1_ESM.docx]

| **Supplementary Table S1.** Discrimination power of liver fibrosis indices and fetuin-A for diabetic peripheral neuropathy | | | | | | |
| --- | --- | --- | --- | --- | --- | --- |
| Test | AUROC  (95% CI) | Cut-off | Sens. (%) | Spec. (%) | PPV  (%) | NPV  (%) |
| All individuals | | | | | | |
| NAFLD fibrosis score | 0.581  (0.510, 0.652) | -2.83 | 98.0 | 5.5 | 39.1 | 81.8 |
|  |  | -1.81 | 86.1 | 27.0 | 42.2 | 75.9 |
|  |  | -0.81 | 54.5 | 59.5 | 45.5 | 67.8 |
|  |  | 0.19 | 19.8 | 91.4 | 58.8 | 64.8 |
|  |  | 1.2 | 5.0 | 98.2 | 62.5 | 62.5 |
| FIB-4 index | 0.589  (0.517, 0.661) | 0.82 | 87.1 | 16.6 | 39.3 | 67.5 |
|  |  | 1.32 | 57.4 | 55.2 | 44.3 | 67.7 |
|  |  | 1.82 | 31.7 | 86.5 | 59.3 | 67.1 |
|  |  | 2.30 | 14.9 | 95.7 | 68.2 | 64.5 |
| Subgroup of NAFLD liver fat score > -0.640^a^ | | | | | | |
| NAFLD fibrosis score | 0.566  (0.481, 0.651) | -2.74 | 95.9 | 6.2 | 40.1 | 70.0 |
|  |  | -1.71 | 81.1 | 28.3 | 42.6 | 69.6 |
|  |  | -0.71 | 48.6 | 60.2 | 44.4 | 64.2 |
|  |  | 0.31 | 17.6 | 97.3 | 81.3 | 64.3 |
| FIB-4 index | 0.592  (0.506, 0.679) | 0.82 | 85.1 | 15.0 | 39.6 | 60.7 |
|  |  | 1.32 | 58.1 | 54.9 | 45.7 | 66.7 |
|  |  | 1.82 | 32.4 | 88.5 | 64.9 | 66.7 |
|  |  | 2.28 | 17.6 | 96.5 | 76.5 | 64.1 |
| Fetuin-A^b^ | 0.500  (0.373, 0.627) | 459.8 | 17.1 | 82.9 | 50.0 | 50.0 |
|  |  | 512.0 | 36.6 | 75.6 | 60.0 | 54.4 |
|  |  | 563.0 | 41.5 | 63.4 | 53.1 | 52.0 |
|  |  | 610.8 | 58.5 | 46.3 | 52.2 | 52.8 |
|  |  | 665.6 | 63.4 | 24.4 | 45.6 | 40.0 |
|  |  | 715.6 | 75.6 | 17.1 | 47.7 | 41.2 |
|  |  | 766.1 | 87.8 | 12.2 | 50.0 | 50.0 |
| ^a^*n* = 187, and ^b^*n* = 82. AUROC, area under the receiver operating characteristic curve; CI, confidence interval; Sens., sensitivity; Spec., specificity; FIB-4, fibrosis-4; PPV, positive predictive value; NPV, negative predictive value. | | | | | | |

**Liver fibrosis indices are related to diabetic peripheral neuropathy in individuals with type 2 diabetes**

Kyuho Kim^1^, Tae Jung Oh^1,3^, Hyen Chung Cho^1^, Yun Kyung Lee^1^, Chang Ho Ahn^1,3^, Bo Kyung Koo^2,3^, Jae Hoon Moon^1,3^, Sung Hee Choi^1,3^, Hak Chul Jang^1,3^
